# The Therapeutic Efficacy of Gold Needle “Regulating Spirit” Acupuncture for Amnestic Mild Cognitive Impairment: Protocol for a Randomized Controlled Trial

**DOI:** 10.2196/96326

**Published:** 2026-08-05

**Authors:** Xin Wang, Yilin Tao, Qianqian Li, Jing Yang, Qian Liu, Anping Xu

**Affiliations:** 1 Department of Psychosomatic Medicine, Beijing Hospital of Traditional Chinese Medicine affiliated to Capital Medical University, Beijing, China; 2 College of Acupuncture-Moxibustion and Tuina, Beijing University of Chinese Medicine, No.11, North Third Ring Road East Chaoyang District, Beijing, 100020, China, 86 01053912199

**Keywords:** Gold Needle "Regulating Spirit", acupuncture, amnesic mild cognitive impairment, randomized controlled trial, Alzheimer disease

## Abstract

**Background:**

Amnestic mild cognitive impairment (aMCI), the predominant subtype of mild cognitive impairment, carries the highest risk of progression to Alzheimer disease among all mild cognitive impairment subtypes. Currently, clinical practice lacks an established, authoritative treatment method for this condition. Prior clinical evidence indicates acupuncture may enhance cognitive function in individuals with mild cognitive impairment. Further clinical evidence suggests Gold Needle therapy demonstrates significant therapeutic effects for challenging and refractory conditions; however, a critical gap exists: no clinical trials currently ascertain whether Gold Needle therapy surpasses conventional acupuncture in treating aMCI. This trial aims to rigorously evaluate the therapeutic efficacy and safety of the Gold Needle “Regulating Spirit” method for clinical symptoms in patients with aMCI, alongside investigating its underlying imaging and biochemical mechanisms.

**Objective:**

This proposed study aims to identify imaging and laboratory biomarkers for the early diagnosis of aMCI, thereby providing a theoretical foundation for clinical practice.

**Methods:**

This randomized controlled trial will recruit 90 patients diagnosed with aMCI from the Beijing Hospital of Traditional Chinese Medicine, affiliated with Capital Medical University, alongside 20 healthy volunteers. The 90 patients with aMCI will be randomly allocated to 1 of 3 groups: the Gold Needle “Regulating Spirit” group uses gold-based needles, the general acupuncture “Regulating Spirit” group, or a sham acupuncture control group. The Gold Needle “Regulate Spirit” group uses gold-based needles, the general acupuncture “Regulate Spirit” group uses standard needles, and the sham acupuncture group administers Park needles. Participants will undergo 3 sessions per week of their assigned acupuncture or placebo treatment over a continuous 12-week period.

**Results:**

This study was initiated on September 1, 2023. As of October 30, 2025, 110 eligible participants had been enrolled, and data collection had been completed in full. Data analysis is currently underway, and the preliminary results are expected to be available by June 2025. We hypothesize that, compared with the filiform needle-based mind-regulating acupuncture group, the golden needle-based mind-regulating acupuncture group will demonstrate superior efficacy in improving cognitive impairment. This superiority will be reflected in multiple key outcome measures, including Montreal Cognitive Assessment and Mini-Mental State Examination scores, plasma biomarkers, and functional magnetic resonance imaging findings.

**Conclusions:**

We anticipate that by the end of the trial, we will be able to definitively ascertain whether the gold acupuncture needle technique for “regulating spirit” offers a significant advantage in treating aMCI, and further investigate the nature of this therapeutic benefit, to offer a more efficacious intervention for the clinical management of aMCI and the prevention of Alzheimer disease.

## Introduction

Mild cognitive impairment (MCI) is characterized as an intermediary condition between normal aging and Alzheimer disease (AD), wherein individuals experience memory deficits that exceed the normative expectations for their age, alongside varying degrees of impairments in other cognitive functions; however, these symptoms do not significantly disrupt daily living activities [1]. Recent epidemiological research suggests that the estimated overall prevalence of MCI in China is approximately 15.5% [2]. As the aging population in China continues to expand, there has been a significant rise in the prevalence of MCI. MCI encompasses several subtypes, with the most prevalent being amnestic mild cognitive impairment (aMCI) [3]. Distinct from other subtypes, aMCI is primarily characterized by a significant decline in memory function [4], which serves as its principal clinical manifestation. This feature renders it more closely associated with AD and makes it the subtype most susceptible to conversion into AD [5]. A study conducted among a random sample of 3246 older Chinese community members revealed that the prevalence of aMCI was 17.1%, with an annual incidence rate of 70.57%. Furthermore, the conversion rate from aMCI to AD in the absence of intervention ranged between 10% and 17% [6]. The most salient manifestations of AD include significant memory deficits, alongside cognitive deterioration, visual-spatial dysfunction, executive function impairment, and neuropsychiatric symptoms that are independent of cognitive processes [7]. These symptoms collectively contribute to a deterioration in the capacity for independent living and a significant impairment of self-care abilities, thereby profoundly impacting the quality of life as well as the physical and mental health of older adults. aMCI is regarded as a preliminary phase of AD, and identifying effective treatment strategies for aMCI holds significant clinical implications for the prevention of AD.

At present, the management of aMCI predominantly relies on pharmacological interventions, with the medications used frequently mirroring those used in AD treatment strategies, including donepezil, memantine, and cholinesterase inhibitors [8,9]. Nevertheless, research has demonstrated that these medications not only fail to impede the progression of aMCI to AD but also induce adverse effects during their administration [10]. Therefore, there exists an urgent clinical demand for a safer alternative treatment with reduced side effects for individuals diagnosed with aMCI. Acupuncture is a traditional Chinese medical practice and one of the primary modalities for disease treatment within traditional Chinese medicine. It is considered safe, exhibits minimal side effects, and demonstrates significant long-term efficacy. Acupuncture has demonstrated potential efficacy in ameliorating mild cognitive impairment and enhancing memory, positioning it as a viable complementary and alternative therapy [11,12]. Our prior animal studies have demonstrated that acupuncture can enhance memory and alleviate cognitive impairments in AD mouse models, thereby offering a substantial experimental foundation [13,14]. Gold Needles, primarily composed of gold, are used in acupuncture treatments due to their advantageous properties. In comparison to other metallic materials, gold exhibits more stable chemical characteristics and a softer texture, facilitating precise control over penetration direction while minimizing discomfort for patients [15]. In clinical practice, the therapeutic efficacy of Gold Needles for a range of conditions, including neurological disorders, surpasses that of conventional metal needles [15]. Currently, there is a paucity of high-quality clinical trials investigating acupuncture treatment for aMCI, and no clinical studies have been conducted on Gold Needle intervention for this condition. Therefore, it is imperative to undertake relevant clinical investigations. The onset of aMCI is insidious, and certain symptoms may not be readily apparent, rendering them susceptible to oversight. As an early manifestation of AD, aMCI exhibits numerous similarities with AD [16]. The precise mechanisms underlying the development of aMCI remain unclear, and numerous hypotheses have been proposed to elucidate this phenomenon. aMCI is predominantly characterized by the development of senile plaques formed from beta-amyloid (Aβ) protein deposits, alongside the accumulation of tau-related proteins that give rise to neurofibrillary tangles [17].

In light of the clinical symptoms and pathological characteristics associated with aMCI, this trial incorporates a comprehensive approach that includes neuropsychological assessments, cognitive function evaluations, imaging analyses, and molecular biological investigations to assess the efficacy and safety of the Gold Needle “regulating the spirit” method in treating aMCI.

## Methods

### Study Design

This study is designed as a randomized controlled trial (RCT) using block randomization. We will recruit 90 participants diagnosed with aMCI and allocate them into 3 groups in a 1:1:1 ratio; specifically, the Gold Needle “Regulating Spirit” group, the general acupuncture “Regulating Spirit” group, and a sham acupuncture control group. Additionally, we will include 20 healthy volunteers to serve as a control group. The participants’ gender, age, and educational background will be aligned with those of the patients with aMCI. A total of 90 patients with aMCI will undergo treatment for 12 weeks in groups, receiving interventions 3 times per week. Data collection for all enrolled individuals will occur at baseline (week 0), week 12, and week 24.

**Figure 1.**
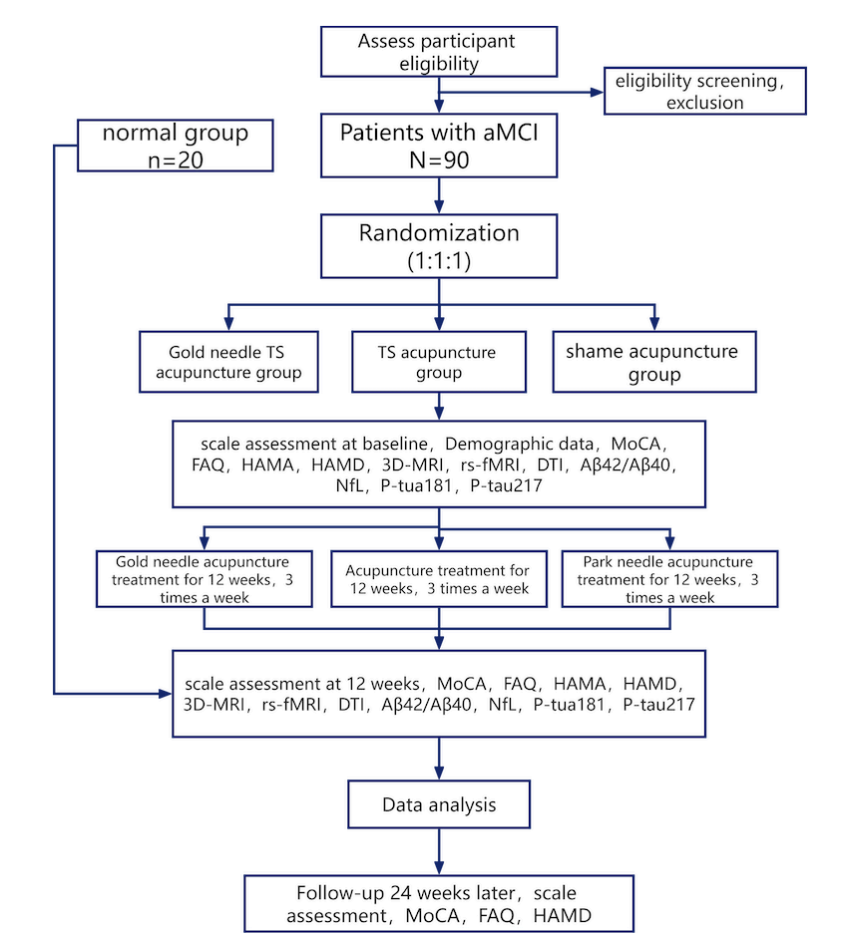
Flowchart diagram. aMCI: amnestic mild cognitive impairment; DTI: diffusion tensor imaging; FAQ: Functional Activities Questionnaire; HAMA: Hamilton Anxiety Scale; HAMD: Hamilton Depression Scale; 3D-MRI: 3D–magnetic resonance imaging; Aβ: beta-amyloid; MoCA: Montreal Cognitive Assessment; NfL: neurofilament light chain; rs-fMRI: resting-state functional magnetic resonance imaging; TS: Tiao shen (English: “Regulating Spirit”).

The study protocol has been registered in ClinicalTrials [18]. The flowchart of the trial is shown in Figure 1 and the research content at different periods of the trial is given in Table 1.

**Table 1.** Research content during different periods of the trial.

	Enrolment	Baseline	Intervention period (12 wk, 3 times per week)				Follow-up
Week	−1	0	1-4	5-8	9-11	12	24
Inclusion criteria	✓						
Exclusion criteria	✓						
Randomization	✓						
Informed consent	✓						
Intervention							
Gold Needle–TS^a^ acupuncture			✓	✓	✓	✓	
TS-acupuncture			✓	✓	✓	✓	
Shame acupuncture			✓	✓	✓	✓	
Assessment							
Demographic data		✓					
MoCA^b^		✓				✓	✓
FAQ^c^		✓				✓	✓
HAMA^d^		✓				✓	✓
HAMD^e^		✓				✓	✓
3D-MRI^f^		✓				✓	
rs-fMRI^g^		✓				✓	
DTI^h^		✓				✓	
Aβ^i^_42_/Aβ_40_		✓				✓	
NfL^j^		✓				✓	
P-tua181		✓				✓	
P-tua217		✓				✓	
Adverse events			✓	✓	✓	✓	
Reasons for dropout			✓	✓	✓	✓	
Withdrawals			✓	✓	✓	✓	

a TS: Tiao shen (English: “Regulating Spirit”).

b MoCA: Montreal Cognitive Assessment.

c FAQ: Functional Activities Questionnaire.

d HAMA: Hamilton Anxiety Scale.

e HAMD: Hamilton Depression Scale.

f 3D-MRI: 3D–magnetic resonance imaging.

g rs-fMRI: resting-state functional magnetic resonance imaging.

h DTI: diffusion tensor imaging.

i Aβ: beta-amyloid

j NfL: neurofilament light chain.

### Recruitment and Study Population

This study used a prospective, RCT design. Individuals with memory impairment were recruited through hospital referrals, poster advertisements, and community screenings conducted by collaborating organizations. Initial screenings identified individuals who met the inclusion criteria for the study’s aMCI cohort. All participants provided voluntary written informed consent prior to the commencement of the study.

### Eligibility

#### Diagnostic Criteria

In accordance with the diagnostic criteria for aMCI established in 2004, participants were required to meet the following criteria: (1) memory impairment as the chief complaint, confirmed by a knowledgeable person; (2) objective evidence of memory impairment, with the observed decline in memory not proportional to age-related expectations; (3) other general cognitive functions essentially normal; (4) cognitive impairment insufficient to warrant a diagnosis of dementia and evaluated in relation to the individual’s prior performance, without notable deficits in social or occupational functioning; and (5) preserved functional independence, with fundamental daily living skills maintained within normal limits.

#### Inclusion Criteria

The inclusion criteria for patients with aMCI were as follows: (1) meeting the diagnostic criteria for aMCI; (2) the initial episode of illness is characterized by a history of memory impairment lasting at least 3 months, with no prior indications of other neurological or psychiatric disorders; (3) Clinical Dementia Rating score of 0.5 points; (4) individuals aged between 50 and 75 years, with no gender restrictions; (5) the minimum educational duration is 1 year or more; and (6) participation was voluntary, and informed consent was obtained from the person participating.

The inclusion criteria for healthy volunteers are as follows. First, the age should be between 50 and 75 years (male or female). Second, there should be no documented history of abnormalities related to the heart, liver, kidneys, digestive tract, nervous system, psychological conditions, and metabolic disorders. A thorough physical examination should reveal normal findings in electrocardiogram readings, blood pressure, heart rate, respiratory function, and pertinent laboratory tests. Finally, there should be no linguistic or cognitive barriers, allowing for straightforward completion of the questionnaire.

#### Exclusion Criteria

Exclusion criteria for patients with aMCI are as follows: (1) a history of prior intracranial hemorrhage or ischemic disease accompanied by neurological localizing signs, along with imaging findings indicative of cerebral small vessel disease (Fazekas score ≥2 points); (2) individuals experiencing various neurological disorders that may lead to brain dysfunction, including but not limited to epilepsy, depression, brain tumors, Parkinson disease, metabolic disorders, encephalitis, multiple sclerosis, head trauma, and normal pressure hydrocephalus; (3) cognitive impairment associated with various systemic diseases, including liver dysfunction, renal dysfunction, thyroid disorders, severe anemia, deficiencies in folic acid and vitamin B_12_, carbon monoxide poisoning, specific infections (such as syphilis and HIV/AIDS), as well as substance use involving alcohol and drugs; (4) a delay in mental and neurological development; (5) certain contraindications for nuclear magnetic resonance examinations; (6) a disease that makes it impossible for a person to complete cognitive tests; (7) within 1 month of initiating treatment with antidementia pharmacological agents; and (8) the participants declined to provide written, informed consent for their involvement in the study.

Exclusion criteria for healthy volunteers are as follows: (1) a well-documented history of disorders affecting the central nervous system, cardiovascular system, respiratory system, kidneys, liver, digestive tract, or other diseases and physiological conditions that may potentially confound test results is evident; (2) consumption of any prescription or over-the-counter medication within 2 weeks prior to the commencement of the experiment; and (3) severe acute or chronic organic and mental health disorders.

### Randomization, Allocation Concealment, and Blinding

This study used a block-randomized design, wherein an independent statistician—who was not involved in trial assessment, treatment allocation, or statistical analysis—generated the randomization sequence using SAS 9.2 (SAS Institute) software’s PROC PLAN (with a block length of 6). An independent research assistant ensured allocation concealment through the use of opaque, sealed envelopes. In this study, the acupuncturist, outcome evaluator, and data analyst were assigned by different individuals to ensure objectivity; both patients and evaluators remained blinded throughout the process. Given the unique nature of Gold Needle manipulation, challenges arose in establishing acupoint settings for the sham group and in maintaining blinding for the acupuncturist. To address these issues, we strictly adhered to patient blinding protocols and scheduled individual appointments at staggered times.

### Intervention

#### Gold Needle “Regulating Spirit” Group

The patients in this study underwent Gold Needle “Regulating Spirit” therapy, which included acupuncture sessions administered three times per week over a duration of 12 weeks. The acupoint locations follow the 2006 version of *Names and Locations of Acupoints* (GB/T12346-2006) and the operational techniques are in accordance with the national standard textbook of *Moxibustion and Acupuncture Techniques*. The selected acupoints include Baihui (GV20), Sishencong (EX-HN1), Shenting (GV24), Benshen (GB13), Yintang (GV29), Renzhong (GV26), Shenmen (HT7), Feishu (BL13), Xinshu (BL15), Ganshu (BL18), Pishu (BL20), Shenshu (BL23), and Geshu (BL17; the locations are presented in the following table). The patient is positioned in laterally exposing the head, back, and wrist areas. Disposable Huatuo brand sterile gold needles 0.30×40 mm. The acupuncture points and the practitioner’s hands are thoroughly disinfected with 75% alcohol. All acupoints on the head are needled at an angle of 15‐30° to a depth of 1 inch, while those on the back of the body are needled at the same angle and depth but directed upward. At GV26, the needle is inserted 0.3 cun upward, while direct insertion at GV29 and HT7 is performed at a depth of 0.3 cun. After obtaining the “Qi,” each acupuncture point is needled for 30 seconds. Using a technique of balanced supplementation and drainage, the needle is retained in situ for 30 minutes until the patient experiences a moderate sensation of soreness or fullness in the affected region. The locations of each acupuncture point are listed in Table 2.

**Table 2.** The acupuncture points and their locations.

Acupoints	Locations
Baihui (GV20)	At the top of the head, 5 cun directly above the anterior hairline. In the depression midway between the anterior and posterior hairline, 1 cun anterior to the midpoint.
Sishencong (EX-HN1)	On the vertex, 1 cun from the front, back, left, and right to GV20, common 4 points
Shenting (GV24)	At the head, 0.5 cun straight up from the midpoint of the front hairline.
Benshen (GB13)	Located at the superior aspect, 0.5 cun above the anterior hairline and 3 cun lateral to the midline of the cranium.
Yintang (GV29)	At the forehead, at the midpoint between the two medial ends of the eyebrow.
Renzhong (GV26)	In the face, at the point where the upper 1/3 of the philtrum meets the middle 1/3.
Shenmen (HT7)	In the anterior region of the forearm, at the distal lateral aspect of the ulnar side of the palmar flexor tendon, adjacent to the radial side of the wrist.
Feishu (BL13)	In the spinal area, 1.5 cun lateral to the midline at the third thoracic spinous process.
Xinshu (BL15)	In the spinal area, at the level of the 5th thoracic vertebra, 1.5 cun lateral to the midline posterior.
Ganshu (BL18)	In the spinal area, at the level of the 9th thoracic vertebra, 1.5 cun lateral to the midline posteriorly.
Pishu (BL20)	In the spinal region, at the level of the 11th thoracic vertebra, 1.5 cun lateral to the midline posteriorly.
Shenshu (BL23)	In the spinal area, 1.5 cun lateral to the midline at the level of the second lumbar spinous process.
Geshu (BL17)	In the spinal area, 1.5 cun lateral to the midline at the 7th thoracic spinous process.

#### The General Acupuncture “Regulating Spirit” Group

These patients received moxibustion therapy using general needles to regulate the spirit, with acupuncture sessions conducted three times a week for a total of 12 weeks. The acupoint locations adhere to the standards established by the World Health Organization (WHO) in 2008, while the operational techniques are based on the national standard textbook titled *Acupuncture and Moxibustion*. The selection of acupoints aligns with that of the Gold Needle “Regulating Spirit” group. Sterile disposable needles from the Huatuo brand, measuring 0.30×40 mm, are used. Position, technique, and manipulation are the same as those in the Gold Needle group.

#### Sham Acupuncture Group

The needle used is the model 0.30×40 mm Park Comfort Needle. The comfort needle is designed to resemble a conventional needle, featuring a collapsible blunt tip. Upon contact with the skin, this blunt tip automatically retracts into the hollow shaft of the needle, preventing any penetration. Acupuncture point manipulation: Feishu (BL13), Xinshu (BL15), Ganshu (BL18), Pishu (BL20), Shenshu (BL23), and Geshu (BL17). Park comfortably administered acupuncture treatments by selecting only back acupoints, strictly adhering to the blinding of patients, and scheduling individual appointments at different times.

#### Normal Group

Participants in the normal group did not require any treatment.

### Outcome Assessment

The variables examined in this study encompass both demographic and clinical characteristics, as well as the primary and secondary outcome measures. The fundamental characteristics of each group were assessed at baseline (wk 0). The primary and secondary outcome measures were evaluated at baseline, at the conclusion of the intervention (wk 12), and again at 24 weeks post follow-up.

### Basic Characteristics

Recruiters will use a self-constructed questionnaire to collect demographic information from participants, including gender, age, educational attainment, marital status, living arrangements, occupation, and socioeconomic status, along with their medical history and substance use.

### Primary Outcomes

The Montreal Cognitive Assessment (MoCA) was used to assess the patients’ overall cognitive function [19]. MoCA encompasses several cognitive domains, including Visuospatial or Executive Function, Attention and Calculation, Naming, Delayed Recall, Orientation, Language, and Abstract Reasoning. The total score ranges from 0 to 30 points, with higher scores indicating superior functionality.

### Secondary Outcomes

Neuropsychiatric symptoms and a decline in functional abilities are significant concomitant manifestations of AD, with their emergence serving as an indicator that aMCI is progressing toward AD [20]. Consequently, this study incorporated the Functional Activities Questionnaire, along with the Hamilton Anxiety Scale and the Hamilton Depression Scale [21,22].

### Other Indicators

Acquire multimodal magnetic resonance imaging (MRI) data, including 3D-MRI, resting-state functional MRI, and diffusion tensor imaging, to construct networks related to cognitive function and brain structure [23]. The study participants, who underwent imaging, were scanned using a Siemens Novus 3.0T MRI system, which was manufactured in Germany.

Using single-molecule immunoarray technology for the detection of biomarkers in plasma, we assess the intervention level of the gold standard concerning the Aβ42/Aβ40 ratio in peripheral plasma, alongside measurements of plasma neurofilament light chain, plasma P-tau181, and plasma P-tau217 [24,25].

### Safety Measurement

Throughout the study, the acupuncturist systematically monitored, documented, and communicated adverse events (AEs) using open-ended inquiries. Within 24 hours of the AE occurrence, it was categorized as either treatment-related—common examples being subcutaneous hematoma, persistent pain postneedling, and syncope—or treatment-unrelated. Follow-up continued until the AE was adequately resolved.

### Sample Size

This study represents the first application of Gold Needle intervention for the treatment of aMCI. Due to the absence of comparable studies in this domain previously, an accurate estimation of the sample size parameters was not feasible. This study seeks to undertake an exploratory RCT. This study took a group of patients with MCI as the research subjects. In accordance with the sample size recommendations for preclinical trials in traditional Chinese medicine and taking into account parameter precision, a total of 90 participants were selected, comprising 30 individuals in the Gold Needle “Regulating Spirit” group, 30 in the general acupuncture “Regulating Spirit” group, and 30 in the sham acupuncture control group.

### Statistical Analysis

Quantitative data were statistically described using SPSS 21.0 software (IBM Corp) and presented as mean (SD; x±s). Three sets of continuous variable statistics were analyzed: Based on the distribution characteristics of the data, comparisons among groups of continuous variables were conducted using either ANOVA or the Kruskal-Wallis H test. In cases where a statistically significant overall difference was identified among the 3 groups, post hoc multiple comparisons were performed using either the least significant difference test or the Nemenyi test. Baseline data, along with data collected at week 12 and follow-up data at 24 weeks, were analyzed using repeated measures ANOVA or generalized linear mixed models tailored for repeated measures.

The brain structure images acquired through MRI will be processed using MATLAB (MathWorks) [26], CAT12 (Computational Anatomy Toolbox; C. Gaser, Structural Brain Mapping Group, University of Jena, Germany) [27], and FSL (FMRI Software Library 5.0; Analysis Group, FMRIB, University of Oxford, United Kingdom). We used 2 distinct analytical approaches, namely voxel-based analysis and tract-based spatial statistics, to assess alterations in total gray and white matter volumes as well as white matter fiber tracts in a patient with aMCI.

Resting-state functional MRI data will be processed using version 2.3 of the WFU_PickAtlas, developed by the Functional MRI Laboratory at Wake Forest University School of Medicine, which is designed to extract the hippocampal region of interest. Use Conn [28] for data preprocessing and statistical analysis. Use the Conn (McGovern Institute for Brain Research) and SPM12 software (Wellcome Centre for Human Neuroimaging) to perform statistical analysis of the results, including functional connectivity, regional homogeneity, and amplitude of low-frequency fluctuations indicators, and visualize the nodes in 3D using the BrainNet Viewer [29] software.

### Ethical Considerations

This study has been approved by the Medical Ethics Committee of Beijing Hospital of Traditional Chinese Medicine Affiliated to Capital Medical University (2023BL02-064-01) and Ethics Committee of Beijing University of Chinese Medicine (2024BZYLL0101). All participants were provided with a written consent form and recruited signed it prior to being into our study.

## Results

This study was initiated on September 1, 2023. As of October 30, 2025, 110 eligible participants had been enrolled, and data collection had been completed in full. Data analysis is currently underway, and the preliminary results are expected to be available by June 2025. We hypothesize that, compared with the general acupuncture “Regulating Spirit” group, the Gold Needle “Regulating Spirit” group will demonstrate superior efficacy in improving cognitive impairment. This superiority will be reflected in multiple key outcome measures, including MoCA and Mini-Mental State Examination scores, plasma biomarkers, and functional MRI findings.

## Discussion

### Expected Findings

AD is the primary cause of dementia, accounting for 50% to 70% of all cases of dementia [30]. As the aging population expands, the prevalence of AD is expected to rise, particularly in low- and middle-income countries. AD has emerged as the fifth leading cause of mortality among individuals aged 65 years and older, serving as a direct contributor to dependency and disability [31]. AD is a neurodegenerative disorder characterized by the progressive and often irreversible degeneration of neurons, with treatment efficacy diminishing as the age of the patient increases, irrespective of the therapeutic interventions used [32]. According to the AD diagnostic guidelines published in the United States, the symptoms of aMCI fulfill the clinical criteria for the predementia stage [33]. Implementing early warning systems for aMCI and ensuring timely intervention during the initial stages of the disease can significantly enhance the prognosis of aMCI. Early screening and diagnosis are essential prerequisites for timely intervention. Given the insidious onset of aMCI, the methodologies used for screening and diagnosis hold critical significance in its management. This study uses screening and diagnostic methodologies that integrate clinical symptoms, localized brain alterations, pathological characteristics, and noninvasive yet convenient detection techniques to deliver a more comprehensive diagnosis and evaluation of the disease from multiple perspectives. While the symptoms of aMCI do not significantly disrupt an individual’s routine daily activities, they are associated with objectively lower performance on cognitive neuropsychological assessments when compared to individuals diagnosed with AD [34]. The clinical manifestations of aMCI can be primarily categorized into 3 domains: cognitive decline, mild deficits in complex work-related daily activities, and noncognitive psychiatric symptoms. The MoCA encompasses a wide array of cognitive domains, enabling the evaluation of an individual’s overall cognitive functioning. The Functional Activities Questionnaire evaluates intricate social competencies and daily living activities. Apathy, depression, anxiety, and atypical nocturnal sleep behaviors are prevalent neuropsychiatric manifestations in individuals with aMCI. To assess these symptoms, the Hamilton Anxiety Scale and the Depression Scale were used. Alterations in brain structure and function represent a significant contributor to aMCI. Imaging modalities offer noninvasive methodologies for assessing the brain’s structural and functional attributes, enabling an analysis of the interplay between these changes. Meanwhile, imaging phenotyping can provide a more accurate and objective basis for the classification and quantification of the disease, further aiding in the search for noninvasive imaging biomarkers for the early diagnosis of aMCI. The detection of biological markers in blood represents a noninvasive and cost-effective approach, with alterations in the levels of associated pathological proteins in plasma closely linked to structural changes in the brain and overall cognitive dysfunction. Furthermore, this method holds significant promise for predicting the transition from normal cognition to MCI [25]. Therefore, this trial used gold acupuncture needles as the primary intervention and used the “adjusting the spirit” acupuncture technique to address aMCI. The study also assessed the efficacy and safety of acupuncture treatment for aMCI through various evaluation methods conducted before and after the intervention. Simultaneously, by integrating imaging techniques with molecular biology, this study evaluates the effects of the Gold Needle “Regulating Spirit” method on the brains of patients with aMCI from multiple perspectives. It aims to deeply investigate the mechanisms underlying acupuncture intervention in aMCI and provide a theoretical foundation for the broader clinical application of Gold Needle “Regulating Spirit” treatment for aMCI, while also identifying potential biomarkers for early clinical diagnosis.

The “regulating spirit” technique, which primarily uses gold acupuncture needles as its therapeutic instrument, uses gold as the principal material. Therapeutic interventions are applied at the Baihui (GV20), Sishencong (EX-HN1), Shenting (GV24), Benshen (GB13), Yintang (GV29), Renzhong (GV26), Shenmen (HT7), Feishu (BL13), Xinshu (BL15), Ganshu (BL18), Pishu (BL20), Shenshu (BL23), and Geshu (BL17). The selection of acupuncture points is grounded in the principles of traditional Chinese medicine. The synergistic application of these acupoints facilitates cognitive clarity, enhances memory retention, and promotes the nourishment of internal organs [35]. The acupoints selected for this trial were determined based on the principles of Diagnosis and Treatment Based on Overall Analysis of the Illness and the Patient’s Condition in traditional Chinese medicine, as well as the clinical practice guidelines established in China. Nevertheless, the scientific evidence supporting these perspectives remains limited, and there is a notable deficiency of high-quality research, resulting in ambiguity regarding the findings—particularly concerning whether the therapeutic effects of gold acupuncture surpass those of conventional acupuncture. This was substantiated through a RCT, establishing a basis for subsequent large-scale RCTs. Due to the inherent characteristics of acupuncture treatment, implementing a blind method poses significant challenges; therefore, we will use a blinded approach to categorize participants into the Gold Needle “Regulating Spirit” group, the general acupuncture “Regulating Spirit” group, and the sham acupuncture group. Considering the placebo effect associated with sham acupuncture, a healthy cohort was designated as the negative control group. The primary challenge of this study lies in the fact that patients with aMCI are typically older, and the manifestations of aMCI tend to be quite subtle, rendering them less conspicuous within the community and not highly regarded by the general population. Moreover, the duration of the intervention for participants with aMCI in this trial was extended, making it crucial to assess their willingness to adhere to treatment throughout the study and their cooperation in the collection of various outcome measures.

### Conclusions

We anticipate that by the end of the trial, we will be able to definitively ascertain whether the gold acupuncture needle technique for “regulating spirit” offers a significant advantage in treating aMCI, and further investigate the nature of this therapeutic benefit, to offer a more efficacious intervention for the clinical management of aMCI and the prevention of AD.

## Supplementary material

10.2196/96326 Peer Review Report 1 Peer review report from the Beijing Municipal Science & Technology Commission No.Z221100007422106) and the Fundamental Research Funds for the Central Universities 2023-JYB-JBQN-029.

## Abbreviations

AD: Alzheimer disease
AE: adverse event
aMCI: amnestic mild cognitive impairment
Aβ: beta-amyloid
CAT12: Computational Anatomy Toolbox
FSL: FMRI Software Library 5.0
MCI: mild cognitive impairment
MoCA: Montreal Cognitive Assessment
MRI: magnetic resonance imaging
RCT: randomized controlled trial
WHO: World Health Organization
