# Supplementary material for: The Therapeutic Efficacy of Gold Needle “Regulating Spirit” Acupuncture for Amnestic Mild Cognitive Impairment: Protocol for a Randomized Controlled Trial
Source: JMIR Res Protoc. 2026 Aug 5;15:e96326. doi: 10.2196/96326 (PMC13440222; doi:10.2196/96326)
Supplement: Supplementary file 1 [file resprot-v15-e96326-s001.pdf]

北京市科技计划课题实施方案专家论证意见

专家组论证意见

课题名称	金针“调神”针法干预遗忘型轻度认知障碍的随机对照试验及临床机制探讨
所属项目名称	首都临床特色诊疗技术研究及转化应用

业务论证意见：

（除《立项细则》正文中规定的论证内容外，科技专家侧重对仪器设备、材料购置及测试加工费的必要性、合理性等提出意见，也可以就经费预算的其他方面提出建议）

该课题应用金针“调神”针法干预遗忘型轻度认知障碍，具有一定的科学意义，该研究采用金针调神组、毫针调神组、假针组治疗遗忘型轻度认知障碍，拟通过认知神经心理学评估与多模态磁共振及生物分子学多个维度进行评价，获取最佳治疗方案，具有一定的临床应用价值。技术路线清晰，组织实施与管理措施合理可行。

课题负责人及其团队具有临床试验基础，研究团队层次合理，经费预算经过调整后合理，具备完成课题的能力。

课题完成后对遗忘型轻度认知障碍患者的治疗，具有一定社会效益及成果推广价值。

经费论证意见：

(财务专家侧重对超出预算支出范围、超标准和支出结构不合理的费用提出核减意见或完善建议，并在专家个人意见表中提出客观、具体的意见或建议）

经费预算调整后基本合理。

试用水印

7.13

北京市科技计划课题实施方案专家论证意见

专家个人意见

课题名称	金针“调神”针法干预遗忘型轻度认知障碍的随机对照试验及临床机制探讨
所属项目名称	首都临床特色诊疗技术研究及转化应用

课题任务和目标、考核指标可行性评价：

课题任务和目标、考核指标可行。

课题研发内容及技术路线、组织实施与管理措施合理性评价：

课题研发内容、技术路线、组织实施与管理措施合理。

课题完成后的经济社会效益分析及成果推广方案评价：

课题完成后的经济社会效益分析及成果推广良好。

课题其他方面的评价或建议：

无

课题经费预算安排合理性评价：

课题经费预算基本合理。

试用水印

北京市科技计划课题实施方案专家论证意见

专家个人意见

课题名称	金针“调神”针法干预遗忘型轻度认知障碍的随机对照试验及临床机制探讨
所属项目名称	首都临床特色诊疗技术研究及转化应用
课题任务和目标、考核指标可行性评价：<br><br>基本可行.	
课题研发内容及技术路线、组织实施与管理措施合理性评价：<br><br>基本合理.	
课题完成后的经济社会效益分析及成果推广方案评价：<br><br>基本可行.	
课题其他方面的评价或建议：<br><br>无	
课题经费预算安排合理性评价：<br><br>基本合理，经费[illegible].	

试用水印
北京市科委计划项目立项论证

北京市科技计划课题实施方案专家论证意见

专家个人意见

课题名称	金针“调神”针法干预遗忘型轻度认知障碍的随机对照试验及临床机制探讨
所属项目名称	首都临床特色诊疗技术研究及转化应用
课题任务和目标、考核指标可行性评价：<br>同意	
课题研发内容及技术路线、组织实施与管理措施合理性评价：<br>同意	
课题完成后的经济社会效益分析及成果推广方案评价：<br>同意	
课题其他方面的评价或建议：<br>无	
课题经费预算安排合理性评价：	

试用水印

北京市科技计划课题实施方案专家论证意见

专家个人意见

课题名称	金针“调神”针法干预遗忘型轻度认知障碍的随机对照试验及临床机制探讨
所属项目名称	首都临床特色诊疗技术研究及转化应用

课题任务和目标、考核指标可行性评价：

该课题任务目标明确，考核指标可行

课题研发内容及技术路线、组织实施与管理措施合理性评价：

课题研发技术路线清晰，组织管理措施合理

课题完成后的经济社会效益分析及成果推广方案评价：

经济社会效益分析合理，成果推广径可行

课题其他方面的评价或建议：

无

课题经费预算安排合理性评价：

合理

试用水印

北京市科技计划课题实施方案专家论证意见

专家个人意见

课题名称	金针“调神”针法干预遗忘型轻度认知障碍的随机对照试验及临床机制探讨
所属项目名称	首都临床特色诊疗技术研究及转化应用
课题任务和目标、考核指标可行性评价：<br>课题任务较明确，考核指标可行	
课题研发内容及技术路线、组织实施与管理措施合理性评价：<br>研发内容和技术路线图清晰，组织实施和管理措施合理	
课题完成后的经济社会效益分析及成果推广方案评价：<br>课题完成后具有一定的经济社会效益，其成果可以推广应用	
课题其他方面的评价或建议：	
课题经费预算安排合理性评价：<br>课题经费预算安排合理	

试用水印

北京市科技计划课题实施方案专家论证意见

专家个人意见

课题名称	金针“调神”针法干预遗忘型轻度认知障碍的随机对照试验及临床机制探讨
所属项目名称	首都临床特色诊疗技术研究及转化应用
课题任务和目标、考核指标可行性评价：<br>针灸治疗 a MCI 的高质量临床试验较少，尚无金针干预 a MCI 的临床研 究，本团队前期相关研究结果证实针刺改善认知功能这一疗效，本研究将认知神经心 理学评估与多模态磁共振评价手段引入金针临床研究中，拟构建a MCI 患者认知相关结构及功能脑网络，探索与健康志愿患者脑网络异同，采用纵向、多时间、重复测量的研究方法，动态提取金针干预前后认知神经心理学评估、影像学表征，深入探讨针刺干预a MCI 机制，为临床推广提供理论依据。	
课题研发内容及技术路线、组织实施与管理措施合理性评价：<br>课题设计合理，技术路线明确，课题研究内容完整可行。	
课题完成后的经济社会效益分析及成果推广方案评价：<br>本研究致力于金针的干预 a MCI 的疗 效评价及金针治疗手段的推广，可能会极大地推广针灸发展，并为早期干预a MCI 预防进展为痴呆提供新的治疗方案。	
课题其他方面的评价或建议：<br>在课题实施期间要加强监管。	
课题经费预算安排合理性评价：<br>经费使用合理	

北京市科技计划课题实施方案专家论证意见

专家个人意见

课题名称	金针“调神”针法干预遗忘型轻度认知障碍的随机对照试验及临床机制探讨
所属项目名称	首都临床特色诊疗技术研究及转化应用
课题任务和目标、考核指标可行性评价：<br>该课题应用金针“调神”针法干预遗忘型轻度认知障碍，具有一定的科学意义，课题任务及目标明确，考核指标可行。	
课题研发内容及技术路线、组织实施与管理措施合理性评价：<br>该研究采用金针调神组、毫针调神组、假针组治疗遗忘型轻度认知障碍，拟通过认知神经心理学评估与多模态磁共振及生物分子学多个维度进行评价，获取最佳治疗方案，具有一定的临床应用价值。技术路线清晰，组织实施与管理措施合理可行。	
课题完成后的经济社会效益分析及成果推广方案评价：<br>课题完成后对遗忘型轻度认知障碍患者的治疗，具有一定社会效益及成果推广价值。	
课题其他方面的评价或建议：<br>无	
课题经费预算安排合理性评价：<br>经费预算调整后基本合理。	
